# An EPID‐based system for gantry‐resolved MLC quality assurance for VMAT

**DOI:** 10.1120/jacmp.v17i5.6312

**Published:** 2016-09-08

**Authors:** Benjamin J. Zwan, Michael P. Barnes, Todsaporn Fuangrod, Cameron J. Stanton, Daryl J. O'Connor, Paul J. Keall, Peter B. Greer

**Affiliations:** ^1^ Central Coast Cancer Centre, Gosford Hospital Gosford NSW Australia; ^2^ School of Mathematical and Physical Sciences, University of Newcastle Newcastle NSW Australia; ^3^ Department of Radiation Oncology Calvary Mater Hospital Newcastle NSW Australia; ^4^ Radiation Physics Laboratory, University of Sydney Sydney NSW Australia; ^5^ University of Newcastle Newcastle NSW Australia

**Keywords:** VMAT, MLC, EPID, QA

## Abstract

Multileaf collimator (MLC) positions should be precisely and independently measured as a function of gantry angle as part of a comprehensive quality assurance (QA) program for volumetric‐modulated arc therapy (VMAT). It is also ideal that such a QA program has the ability to relate MLC positional accuracy to patient‐specific dosimetry in order to determine the clinical significance of any detected MLC errors. In this work we propose a method to verify individual MLC trajectories during VMAT deliveries for use as a routine linear accelerator QA tool. We also extend this method to reconstruct the 3D patient dose in the treatment planning system based on the measured MLC trajectories and the original DICOM plan file. The method relies on extracting MLC positions from EPID images acquired at 8.41 fps during clinical VMAT deliveries. A gantry angle is automatically tagged to each image in order to obtain the MLC trajectories as a function of gantry angle. This analysis was performed for six clinical VMAT plans acquired at monthly intervals for three months. The measured trajectories for each delivery were compared to the MLC positions from the DICOM plan file. The maximum mean error detected was 0.07 mm and a maximum root‐mean‐square error was 0.8 mm for any leaf of any delivery. The sensitivity of this system was characterized by introducing random and systematic MLC errors into the test plans. It was demonstrated that the system is capable of detecting random and systematic errors on the range of 1–2 mm and single leaf calibration errors of 0.5 mm. The methodology developed in the work has potential to be used for efficient routine linear accelerator MLC QA and pretreatment patient‐specific QA and has the ability to relate measured MLC positional errors to 3D dosimetric errors within a patient volume.

PACS number(s): 87.55.Qr

## I. INTRODUCTION

Volumetric‐modulated arc therapy (VMAT) is a modern delivery technique in radiotherapy, in which a precise three‐dimensional dose distribution is achieved by delivering a spatially modulated photon beam as the gantry is rotated through one or more arcs.^1^, ^2^, ^3^ Spatial modulation is achieved by means of dynamic multileaf collimator (MLC) trajectories which are synchronized to the gantry angle and dose rate (machine output). These three dynamic components (i.e., MLC, gantry angle, and dose rate) must remain synchronized throughout the treatment in order to deliver the intended dose to the planning target volume (PTV) and organs at risk (OAR). Due to this high level of complexity, there is a need for comprehensive and informative quality assurance (QA) techniques.^4^, ^5^

Positional MLC errors can result in substantial dose differences during VMAT delivery, and this becomes more apparent for highly modulated fields, where the distance between opposing MLC leaves is often small. A number of groups have investigated the dosimetric impact of different types of MLC errors in IMRT and VMAT deliveries.^6^, ^7^, ^8^, ^9^, ^10^, ^11^, ^12^ Rangel and Dunscombe^(12)^ estimated that every 1 mm of systematic shift in MLC positions results in a difference of 2.7% and 5.6% to the reference equivalent uniform dose for prostate and head and neck IMRT fields, respectively. Oliver et al.^(10)^ reported the sensitivity of VMAT deliveries to both random and systematic MLC errors. For systematic errors, dose differences as large as 2.8 Gy per mm of positional error were observed over the course of a treatment.^(10)^

A comprehensive VMAT MLC positioning test should:

- provide an accurate and quantitative determination of MLC positioning versus gantry angle
- be an independent measure of the linear accelerator MLC positioning (i.e., not be based on the linacs readout systems)
- be efficient enough to perform on a regular basis
- use clinically meaningful MLC deliveries
- be able to relate the measured positioning errors to clinical significance (i.e., dose delivered to the patient)

Despite the demonstrated dosimetric significance of VMAT MLC errors, MLC positioning QA for VMAT is still generally performed in a qualitative manner. The common tests employed for VMAT linac QA are the tests proposed by Ling et al.^(4)^ as these are straightforward to deliver and analyze qualitatively. However, the tests proposed by Ling and colleagues were never intended as a comprehensive test system for VMAT deliveries and do not fulfill the above criteria. The Ling MLC positioning test for VMAT is a modification of the well‐known Picket Fence pattern developed for IMRT. In this test, the leaves move in a single direction, a uniform leaf speed is used, and the leaf positions are not determined quantitatively. A set of different tests were proposed by van Esch et al.;^(5)^ however, these are very time‐consuming, involve film dosimetry, and have not gained widespread use. More recently, the Netherlands Commission on Radiation Dosimetry has proposed a more comprehensive quality assurance program for VMAT.^(13)^ It emphasizes that the MLC position as a function of gantry angle should be assessed during dynamic gantry rotation and with dynamic MLC leaf trajectories.

An attractive option for efficient QA is to use the dynamic log files (DynaLog files). These files record the position of each MLC leaf throughout the delivery as measured by the MLC motor encoders and have been used extensively for MLC position testing.^4^, ^14^, ^15^, ^16^, ^17^, ^18^, ^19^, ^20^ Other studies have extended this further and used DynaLog log files to relate the actual MLC position to the dose delivered to patient for IMRT and VMAT patient‐specific QA and delivery verification.^21^, ^22^, ^23^, ^24^ Schreibmann et al.^(23)^ used DynaLog‐recorded MLC positions to create a DICOM‐compliant plan which could be imported into the treatment planning system to perform a 3D dose reconstruction. The use of DynaLog files for MLC QA offers a number of advantages, such as the ability to streamline and automate QA processes; however, due the fact that measured MLC positions are sourced from the MLC controller itself, these measurements are not independent. As a result many types of MLC errors, such as miscalibration errors or mechanical backlash, cannot be detected by analyzing these files, as demonstrated by Agnew et al.^(14)^ This kind of approach should therefore not be used for VMAT linac QA.

To perform VMAT MLC positioning quality assurance, therefore, requires a detector with sufficient resolution both temporally and spatially. Groups have also investigated the use of 2D detector array devices such as MapCHECK, MatriXX, and PTW‐729^25^, ^26^ to assess dynamic MLC performance; however, the applicability of these devices for MLC QA is fundamentally limited by their low spatial resolution. Equipment setup and analysis time can also be significant.

Electronic portal imaging devices (EPIDs) represent a nearly ideal detector for this purpose. They have high spatial resolution (∼0.3mm) and temporal resolution (∼10Hz) with large surface area and minimal setup time. EPIDs have also been employed to assess MLC performance using dynamic MLC test patterns,^8^, ^14^, ^27^, ^28^, ^29^, ^30^, ^31^ as well as for clinical IMRT fields.^8^, ^32^, ^33^, ^34^, ^35^, ^36^, ^37^, ^38^ Some authors have investigated using the EPID to detect MLC positions during VMAT deliveries, and compared these to planned MLC positions for patient‐specific quality assurance^36^, ^37^, ^39^, ^40^ purposes. However the precision of these tests were not adequate for routine MLC positioning quality assurance. For MLC quality assurance Rowshanfarzad et al.^(27)^ investigated the behavior of the MLC during the delivery of dynamic sliding gap fields under a variety of conditions, including both static gantry and arc‐type deliveries, but not absolute MLC position. There is therefore a need to develop a high‐precision method to measure MLC position versus gantry angle in an efficient manner which will form an important component of a comprehensive VMAT quality assurance program.

In this work, a method is presented where EPID image frames are used to measure MLC leaf positions as a function of gantry angle during clinical VMAT deliveries. The method is a very high precision and efficient QA technique that fulfills the criteria listed above for VMAT MLC positioning quality assurance and the testing outlined in the recent Netherlands Report 24.^(13)^ The method requires no knowledge of the planned delivery and uses no information from the treatment delivery system and, therefore, can be used as a completely independent verification tool. The measured MLC positions are compared geometrically to the planned MLC positions and are also written into a new DICOM plan file which can be used to recalculate the 3D dose using a conventional treatment planning system. The sensitivity of the QA tool to detect clinically meaningful MLC positioning errors is demonstrated.

## II. MATERIALS AND METHODS

### A. Delivery system and EPID image acquisition

All measurements in this study were performed using a Varian Trilogy linear accelerator with a 6 MV photon beam. EPID images were collected using a Varian aS1000 EPID (Varian Medical Systems, Palo Alto, CA) which was operated in integrated acquisition mode. Images were performed with a source to detector distance (SDD) of 150 cm, which improves the spatial resolution of the images. Individual image frames were collected at a rate of 8.41 frames per second (fps) using a frame grabber system and in‐house acquisition software written in MATLAB programming language (MathWorks, Natick, MA). Each EPID image frame was automatically flood field and dark field corrected and image acquisition and analysis was performed on an ancillary computer so as not to interfere with the clinical system. All EPID measurements were acquired “in‐air” (i.e., without the presence of a patient or phantom).

### B. Gantry angle measurements

During VMAT deliveries, the gantry angle was determined by accessing information from the gantry angle encoder in the OBI system. Raw encoder‐generated signal was calibrated to gantry angle by varying the gantry angle on the linac console in steps of 45° and recording the encoder signal at each point. Note that, prior to performing these measurements, the machine gantry angle readout from the linac console was independently verified at each cardinal angle using a spirit level. These measurements were repeated for clockwise and counterclockwise rotations and a linear fit was used to model the relationship, as in Woodruff et al.^(41)^

The encoder signal was extracted during delivery from the header of the kilovoltage (kV) image frames, which were acquired simultaneously to the EPID frames, using the dual‐channel functionality of the frame grabber system. The kV image frames were acquired at a frame rate of 15 fps and with the kV source off ensuring no additional dose is delivered to the patient (if present). Both the source and the image panel were retracted during the delivery, so as not to interfere with standard clinical workflow. The encoder‐generated gantry angle was then linearly interpolated as a function of time to tag a gantry angle to each measured EPID image frame. Note that this is necessary due to the different frame rates of the MV and kV imaging systems.

For the Varian Trilogy linac design, the OBI gantry angle encoder operates independently of the delivery system, hence providing a more independent gantry angle measurement than using the imager header gantry angle. Furthermore, the gantry angle tagged to the OBI frame header is highly accurate ($\pm 0.05^{\circ }$) as it is also required for cone‐beam CT reconstructions.^(41)^

### C. MLC position extraction

Each EPID image frame was postprocessed after delivery in order to accurately extract the positions of the in‐field MLC leaves. There are four key steps in this process: mechanical sag correction, collimator rotation measurement, leaf edge detection, and EPID radiation field offset (ERFO) correction.

#### C.1 Mechanical sag correction

As the multiton gantry rotates through a treatment arc, the EPID imaging panel, gantry head, and MLC leaves all experience mechanical sagging effects due to gravity. The magnitude and direction of this sag varies as a function of gantry angle.^(42)^ As a result the isocenter of the EPID imaging panel is not coincident with that of the MLC leaves. In terms of extracting MLC positions, this effect must be compensated for or it will introduce a gantry angle‐dependent accuracy into the QA method, thus producing a measurement bias that can be misleading when interpreting results.

**Figure 1:**
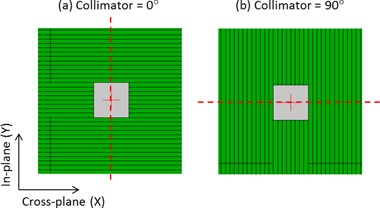
MLC defined fields used to determine the sag characteristics as a function of gantry angle for in the (a) in‐plane direction and (b) cross‐plane direction. These fields were delivered whilst the gantry was rotated from $-180^{\circ }$ to $+180^{\circ }$. The vertical profile for field (a) was used to determine the central axis of the field for the in‐plane direction. The horizontal profile for field (b) was used to determine the central axis of the field in the cross‐plane direction.

**Figure 2:**
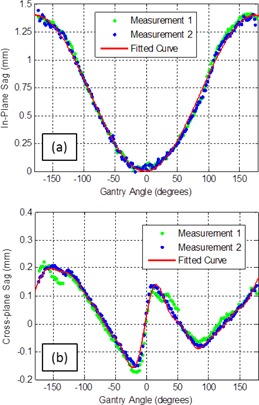
Two measurements of the gantry angle‐dependent sag correction factors for EPID images in the (a) in‐plane and (b) cross‐plane direction. These measurements correspond to static MLC defined fields (see Fig. 1) for gantry rotation in the counterclockwise direction. The two datasets displayed here were measured on the same linac six months apart. The solid line corresponds to a fitted general Fourier curve of degree 1 and degree 8, respectively, for the in‐plane (a) and cross‐plane (b) directions.

In this work, a method has been developed to directly measure the difference between the imager isocenter and MLC isocenter as a function of gantry angle (for the in‐plane and cross‐plane directions). This isocenter correction can then be applied as a 2D shift to acquired images of clinical fields to correct for mechanical sag effects. To determine this correction, a static MLC defined $5\times 5{\text{cm}}^{2}$ radiation field was delivered whilst the gantry was rotated through a full 360° arc and EPID image frames were acquired as a function of gantry angle. This measurement was repeated for 0° and 90° collimator rotation. Using these frame sets, the isocenter shift was determined in the in‐plane and cross‐plane directions, respectively, by detecting the radiation field edge perpendicular to the direction of leaf motion, as indicated in Fig. 1. By using both the 0° and 90° collimator deliveries (rather than a single delivery at 0° collimator rotation), the sag correction can be determined without relying on the actual MLC positions. The mechanical sag was measured as a function of gantry angle using this technique. Figure 2 displays the sag properties for a counterclockwise rotation for two measurements performed on the same linac six months apart. The fitted Fourier curves in Fig. 2 were used to subsequently correct the acquired EPID images.

#### C.2 Collimator rotation measurement

The collimator angle was extracted from each EPID image frame, and subsequently used to rotate the image to represent a field with 0° collimator rotation. This step was performed purely for image processing purpose, so that profile through the central axis of each leaf could be identified. The angle was measured by analyzing the periodic interleaf leakage signal, which forms a sharp peak in the Fourier Transform image. The angle that this peak makes with the origin, in the frequency domain, corresponds to the measured collimator angle. Using this method, the collimator angle can be found to within$\pm 1^{\circ }$ of the planned angle. This method is outlined in greater detail by Fuangrod et al.,^(38)^ who also characterized the accuracy of the technique.

By performing steps as outlined above (mechanical sag correction and collimator rotation measurement), it was ensured that each EPID image is in the same translational and rotational coordinate system as the MLC. This results in a higher geometric accuracy in the MLC position extraction process and allows quality assurance of the X direction leaf motion independent of other components (such as sag and collimator angle accuracy).

#### C.3 MLC leaf edge detection

In order to determine the positions of the MLC leaves in each image, the horizontal profiles through the central axis of each leaf pair were first extracted. Each leaf pair was then classified as either open, closed, or out‐of‐field by comparing the maximum of each profile to the global maximum of the image. For each in‐field leaf pair, two regions of interest were first identified by locating the maximum and minimum gradients of the corresponding profile. Cubic‐spline interpolation was then applied to each identified region of interest in order to determine the coordinates of the 50% radiation field edge to subpixel accuracy. This analysis was repeated for the three central profiles of each leaf, and the mean of these three detected leaf positions was recorded as the final detected field edge. Unless otherwise stated, all measured MLC position in this work refer to the projected positions at the machine isocenter plane.

#### C.4 EPID radiation field offset (ERFO) correction

**Figure 3:**
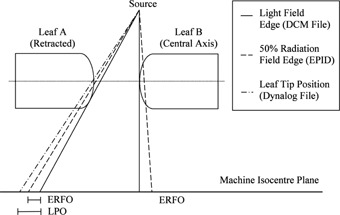
Conceptual diagram outlining the differences between the light field edge (recorded in the DICOM MLC file), 50% radiation field edge (measured by the EPID), and leaf tip position (recorded by the DynaLog file) when projected to the machine isocenter for the rounded leaf tip MLC design. These fundamental differences give rise to a leaf position offset (LPO) and EPID radiation field offset (ERFO).

In order to compare the EPID‐measured MLC field edges with the MLC positions specified in the DICOM plan file an additional correction factor was required. The reason for this is that the EPID‐MLC positions correspond to the 50% intensity field edge; however, MLC positions recorded in the DICOM file correspond to the light field edge (see Fig. 3) as reported by Vial et al.^(43)^ The difference between the light field edge and the 50% radiation field edge is referred to as the Radiation Field Offset (RFO) and is a result of radiation transmission through the rounded leaf tip of the Varian Millennium MLC. The optimal value of the RFO is dependent on a number of factors, most notably the amount of buildup, with larger RFO values corresponding to greater depths. Vial and colleagues characterized this in terms of dose to water for dynamic and static MLC deliveries.

In this work, we present a method for converting measured EPID field edge positions to the light field positions specified by the DICOM plan file using an empirically determined RFO‐like correction factor. Note that the magnitude of the RFO correction for dose measured in water will differ compared the RFO required for EPID measured fields; however, the two corrections are conceptually the same. The EPID‐RFO (ERFO) factor was found by acquiring EPID images of a set of static MLC defined rectangular fields. Each field consisted of a rectangular field centered at a different off‐axis position (ranging from −9cm up to +9cm in steps of 2 cm). Each field was acquired three times, and the MLC was fully retracted between each measured field to avoid any dependence on initial leaf positions. The measurements were performed at 0° gantry and collimator angle and at an SDD of 150 cm.

**Figure 4:**
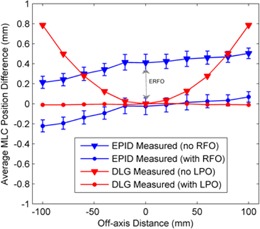
Measured differences between the planned, EPID‐measured, and DynaLog‐measured MLC positions as a function of off‐axis distance. These measurements were used to determine the ERFO correction factor to compare EPID‐measured MLC positions to MLC positions from the DICOM plan file.

Using these data, the planned and measured MLC positions were compared as a function of off‐axis distance for static fields. The ERFO correction factor was defined as the value which minimized the difference between the measured and planned MLC positions for all off‐axis distances. Using these measurements, the optimal ERFO was found to be 0.44 mm (see Fig. 4), which was subsequently used to convert all measured EPID 50% field edges to light field positions prior to comparison with the planned MLC positions from the DICOM plan file. Note that this process was repeated for two separate linear accelerators with identical MLC models and imagers, yielding similar results. Prior to performing these measurements, the positioning accuracy of the MLC was verified on each linac by means of a Picket Fence test using the methodology developed by Rowshanfarzad et al.^(27)^

During acquisition of the static MLC‐defined fields, MLC positions from acquired DynaLog files (Varian Medical Systems) were also recorded and compared to the planned positions from the DICOM file. The DynaLog file positions correspond to the leaf tip positions, which represent the physical edge of the leaves in the central plane of the MLC, as shown in Fig. 4. The difference between the DynaLog measured positions and the DICOM plan file positions is referred to as the leaf position offset^(43)^ (LPO) and varies nonlinearly with off‐axis distance. The MLC control system corrects for this using a table of LPOs which are determined geometrically from the curvature of the MLC leaf tips. Figure 4 shows a comparison between the DynaLog‐measured MLC positions and the planned MLC positions with and without an applied LPO correction for the static test fields.

### D. Dose reconstruction in treatment planning system

The measured MLC trajectories and associated gantry angles were then used to reconstruct the dose in the patient planning CT using an Eclipse TPS (Varian Medical Systems) for clinical VMAT plans. This was done by overwriting the planned MLC positions with the measured positions at each control point in the DICOM RT file. The new “EPID‐altered” DICOM file could then be imported back into an Eclipse TPS which was used to compute the dose in 3D. The planned dose distributions were then compared to the reconstructed dose from the measured MLC positions using a DVH analysis for the planning target volume (PTV) and organs at risk (OAR). The creation of the EPID‐altered DICOM was automated as part of the patient‐specific QA process.

### E. VMAT test fields

The MLC trajectory extraction algorithm was applied to six clinical VMAT test fields consisting of three prostate and three head and neck deliveries. For each case the MLC positions were extracted as a function of gantry angle and were compared to the planned MLC positions from the DICOM RT file. Here, the planned MLC positions were linearly interpolated between control points to obtain the planned MLC positions at the gantry angles corresponding to the measured EPID image frames. The measurement and analysis of each field was repeated three times at monthly intervals to quantify the accuracy and reproducibility of the method. These measurements were performed without the presence of a patient or phantom.

### F. Simulation and detection of MLC positional errors

In order to demonstrate the ability of this system to detect and classify MLC errors, a number of leaf error types were deliberately introduced into a clinical prostate test delivery. This was achieved by altering the MLC positions of the DICOM RT file which could then be delivered in the clinical treatment system. For the purposes of this study, four types of MLC errors were introduced: individual MLC calibration errors, leaf‐pair malfunction errors, random leaf calibration errors, and systematic leaf gap errors.

For individual MLC calibration errors, a single leaf was displaced by a set amount during the entire VMAT delivery. This simulates the occurrence of an isolated leaf miscalibration during a VMAT treatment. The error was introduced into leaf 30 for this particular delivery as this leaf was highly modulated and close to the center of the field and target volume. The detection of this type of error was tested for miscalibrations of 0.25, 0.5, 1, and 2 mm.

Random leaf calibration errors can be described as a miscalibration of every infield MLC leaf by a random amount during the delivery. This imitates the effect of leaf‐calibration walk‐off which can occur if the MLCs are not reinitialized on a regular basis. To simulate this, each leaf was displaced by an amount randomly selected from 0 mm, ±1mm, and ±2mm.

For systematic leaf gap errors, the MLC leaves in each bank were shifted in opposite directions to either create a smaller or a larger leaf gap between each leaf pair. This type of error would be typical of a RFO error as discussed Materials and Methods section C.4 (also referred to as a dosimetric leaf gap error in the Varian Eclipse TPS). Leaf gap errors were simulated at magnitudes of ±1mm and ±2mm, where positive and negative values correspond to larger and smaller gaps, respectively.

For each delivery error test, the dose was then recalculated based on the measured MLC trajectories (Materials and Methods section D). The reconstructed dose was compared to the planned dose in order to indicate the clinical significance of the different error types to each specific patient. The differences between the planned and reconstructed 3D dose distributions were quantified using a DVH analysis of the PTV and relevant OARs.

These error types were chosen as they simulate realistic MLC errors which could potentially be undetectable by other Dynamic MLC QA methods. Note that all of these errors were tested under the same setup conditions and were all introduced into the same VMAT plan, which was previously used as the first prostate test plan (Prostate 1).

## III. RESULTS

### A. Clinical VMAT test plans

**Figure 5:**
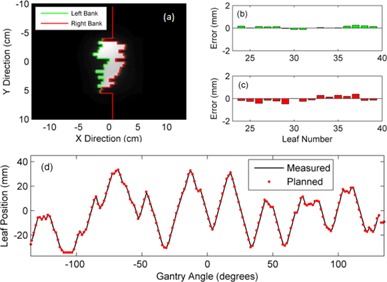
A measured beam aperture (a) from an EPID image frame. The planned MLC positions at the corresponding gantry angle have been superimposed on the grey‐scale EPID image. (b) The difference between the planned and measured MLC positions at this measured EPID image for the left bank and (c) the right bank. (d) A measured single leaf trajectory compared to the planned leaf trajectory. The solid line represents the EPID measured trajectory and the points show the MLC position specified at each control point.

The MLC QA method was tested using three prostate and three head and neck clinical VMAT fields plans, which were used as a sample set to demonstrate the accuracy and functionality of the method. Figure 5 gives an example of the analysis for one of these test fields. It displays the software output for a single aperture ((a) to (c)) of the VMAT delivery, as well as for a single leaf trajectory (d) throughout the VMAT delivery. The software developed in this work has the ability to visualize and verify the MLC aperture at every measured EPID image frame (approximately every 120 ms), as well as verify the trajectory of each individual MLC leaf.

For each of the six test fields, the MLC positions were extracted from the EPID image frames and compared to the planned positions defined by the VMAT control points. The difference between the planned and measured positions are summarized in Table 1, which gives the root‐mean‐square (RMS) error, the mean difference (μ) and the standard deviation (SD) of all in‐field MLC leaves in each delivery.

**Table 1:** Summary of the differences between the planned and measured MLC positions for test deliveries. The difference is averaged for all infield leaves at all measured EPID image frames during the delivery and is given in the form of an RMS error and mean difference, μ±1SD. Each field was measured and analyzed three times, measured at monthly intervals. All measurements are stated in mm.

	*Test 1*		*Test 2*		*Test 3*	
*VMAT Plan*	*RMS*	μ±1SD	*RMS*	μ±1SD	*RMS*	μ±1SD
Prostate 1	0.38	0.06±0.38	0.39	0.07±0.38	0.38	0.05±0.37
Prostate 2	0.39	0.06±0.39	0.38	0.05±0.38	0.40	0.06±0.39
Prostate 3	0.37	0.05±0.37	0.37	0.06±0.37	0.37	0.06±0.38
Head Neck 1	0.44	0.07±0.44	0.45	0.06±0.44	0.45	0.08±0.45
Head Neck 2	0.48	0.03±0.47	0.47	0.04±0.47	0.45	0.04±0.44
Head Neck 3	0.55	0.06±0.54	0.58	0.06±0.58	0.51	0.06±0.50

**Figure 6:**
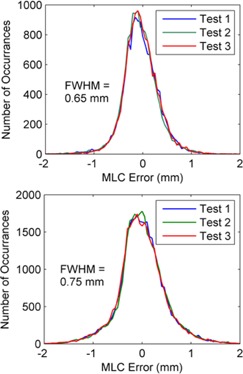
Histogram plot showing the number of detected errors on the vertical axis and the magnitude (and direction) of MLC positional differences on the horizontal axis. Results are displayed for all three test deliveries of the Prostate 1 plan in (a) and Head and Neck 1 plan in (b). The bin width for the histogram plots was 0.05 mm.

The MLC leaf accuracy during a delivery can also be summarized visually using a histogram of the difference between the measured and planned MLC positions. Examples of such histograms are given below for the Prostate 1 and Head Neck 1 test plans in Figs. 6(a) and (b), respectively. A histogram is plotted for each of the three test deliveries.

**Figure 7:**
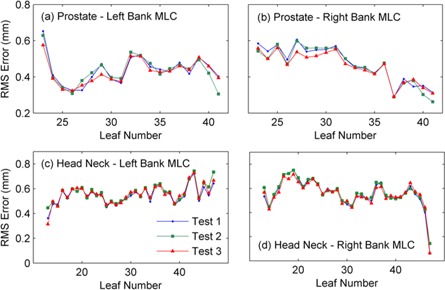
The measured RMS error for each in‐field MLC leaf for the three test deliveries of the Prostate 1 and Head Neck 1 clinical test plans. Results are displayed for the left and right MLC banks for the two test plans.

MLC performance was also analyzed on a leaf‐by‐leaf basis, by computing the RMS position difference (from planned) for each leaf averaged over the entire delivery. Figure 7 details this for the three deliveries of the Prostate 1 test plan and Head Neck 1 test plan.

Additional QA was performed on the Prostate 1 and Head Neck 1 VMAT test plans, where the 3D patient dose was reconstructed in the TPS using the measured MLC positions. The delivered and planned 3D dose distributions were then compared using volumetrically using a DVH analysis of the PTV and relevant OARs, as shown in Table 2.

**Table 2:** DVH comparison between the planned dose distribution and the reconstructed dose distribution, using measured MLC positions, for the Prostate 1 and Head Neck 1 test plans.

*Plan/Volume*	*Plan (Gy)*	*Test 1 (Gy)*	*Test 2 (Gy)*	*Test 3 (Gy)*	*Mean Diff. (%)*
*Prostate 1*					
PTV ($D_{\text{mean}}$)	79.6	79.7	79.7	79.7	0.1
PTV ($D_{\text{max}}$)	83.5	83.9	83.9	83.9	0.5
PTV ($D_{95\%}$)	77	77.1	77.1	77	0.1
Bladder ($D_{\text{mean}}$)	12.9	12.8	12.9	12.8	−0.5
Bladder ($D_{\text{max}}$)	82.8	82.9	83	83	0.2
Rectum ($D_{\text{mean}}$)	40.5	40.7	40.7	40.7	0.5
Rectum ($D_{\text{max}}$)	81.4	81.2	81.2	81.2	−0.2
*Head Neck 1*					
PTV ($D_{\text{mean}}$)	70.8	71.1	71.1	71.1	0.4
PTV ($D_{\text{max}}$)	75.3	75.7	75.8	75.8	0.6
PTV ($D_{95\%}$)	67.3	67.6	67.7	67.6	0.5
Spinal Cord ($D_{\text{max}}$)	34	34.5	34.5	34.5	1.5
Left Parotid ($D_{\text{mean}}$)	23.3	23.4	23.5	23.4	0.6
Left Parotid ($D_{\text{max}}$)	61.1	61.3	61.4	61.2	0.3

### B. Detection of MLC errors in VMAT deliveries

MLC errors were deliberately introduced into a clinical VMAT delivery by varying the MLC positions in the DCM plan file prior to measurement. These errors were introduced into the Prostate 1 test plan (see Table 1) as a case study/example. The measured MLC positions from the altered delivery were then compared to the original planned MLC positions in order to realistically simulate how an error would be detected. The measured MLC trajectories were then used to recalculate the 3D dose distribution, using the method outlined in Materials and Methods D.

#### B.1 Individual leaf calibration errors

**Figure 8:**
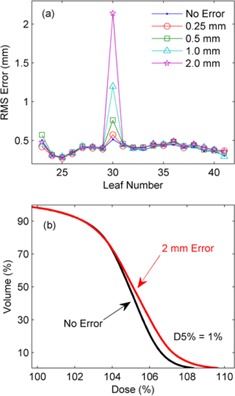
The RMS error (a) for each leaf in the right MLC bank during the delivery for leaf miscalibrations of 0.25, 0.5, 1, and 2 mm. (b) The DVH for the planned PTV and for a 2 mm leaf calibration error.

Figure 8(a) shows the RMS error of each leaf in the right MLC bank during the delivery for leaf miscalibrations of 0.25, 0.5, 1, and 2 mm. The RMS leaf error with no introduced error is also plotted on the same axis for reference. Figure 8(b) displays a DVH analysis for the 2 mm leaf error for the PTV. The DVH for the planned PTV dose is also plotted for reference, yielding a 1% increase in $D_{5\%}$ as a result of the error.

#### B.2 Random leaf calibration errors

**Figure 9:**
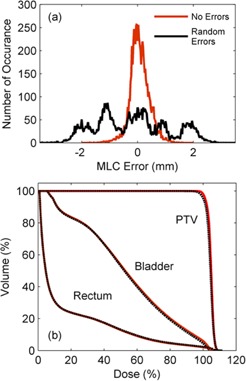
Histogram (a) of leaf position errors for a delivery containing no errors and random MLC calibration errors. The recalculated DVH is also given (b) for the delivery with the random MLC errors, as well as the DVH for the planned delivery. Note that the recalculated DVH for the no errors delivery is not given here as it is visually identical to the original treatment plan.

Random MLC calibration errors were introduced into the test prostate VMAT plan. Figure 9 shows an error histogram of the measured MLC errors for (a) the delivery with no introduced errors and (b) the delivery with random MLC errors. Figure 9(c) plots the DVH for the reconstructed dose with and without errors, indicating the effect of the random MLC errors on the patient dose distribution.

#### B.3 Systematic leaf gap errors

**Figure 10:**
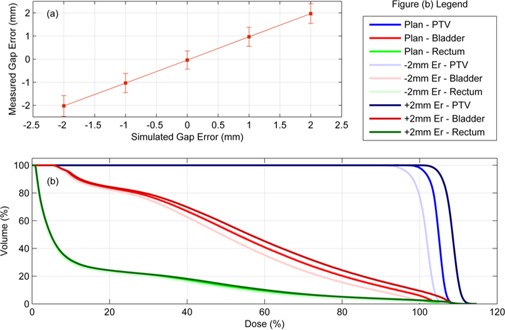
A plot (a) of the measured mean leaf gap error for each leaf gap error that was introduced (0 mm, ±1mm,±2mm). The dose was reconstructed for each case and the DVH is plotted in (b) for the planned dose and±2mm leaf gap error deliveries.

Leaf gap errors of ±1mm and ±2mm were tested. To identify this type of MLC error the planned leaf gap was compared to the measured leaf gap for all MLC leaves, which enabled the computation of the mean and standard deviation of the leaf gap error for the entire delivery. The mean leaf gap errors are plotted in Fig. 10(a) as a function of the actual introduced leaf gap error. The error bars in this plot correspond to one standard deviation (1 SD) of the measured gap errors. Figure 10(b) displays the DVH analysis for each of the ±2mm gap errors as well as no error as a reference.

## IV. DISCUSSION

The complexity of VMAT deliveries necessitates more informative and independent verification of MLC trajectories. It is also ideal to relate MLC positioning accuracy to the dose delivery errors in the patient, so that decisions on MLC performance can be based off metrics that are clinically relevant for the patient. This is not currently possibly using the widely accepted test MLC patterns for arc therapy MLC QA.^4^, ^5^ In this paper the method presented has the ability to directly measure MLC positions during clinical VMAT deliveries. We have also extended this method to reconstruct the 3D dose in the patient using the measured MLC trajectories.

Bakhtiari et al.^(40)^ developed a technique which relied on cine EPID images to assess the MLC positional accuracy during clinical VMAT deliveries. This work successfully demonstrated the feasibility of using gantry angle resolved EPID images for MLC QA. For each leaf of each image a 38% isodose line was used to represent the measured leaf positions and was subsequently compared to the planned positions using a DTA analysis. In this paper we have improved on the method developed by Bakhtiari et al. in a number of ways. Firstly, rather than use the gantry angle from the header of each cine EPID image, which has been shown to have large inaccuracies,^44^, ^45^ we have assigned a gantry angle to each image from encoder of the OBI system which has higher accuracy. We have also implemented a mechanical sag correction to improve the accuracy of the MLC position extraction (1, 2). Another addition is the development of the ERFO correction to compensate for transmission through the leaf tip (4, 5). Using this correction, the planned and measured leaf positions can be directly compared. This enables a further extension of Bakhtiari's geometric QA to use the measured MLC positions to reconstruct the dose delivered to the patient, thus bridging the gap between measured MLC performance and the accuracy of dose delivery. Also note that the ERFO correction was empirically optimized using a 50% dose edge, whilst Bakhtiari and colleagues used a 38% isodose. The difference between these two values is likely compensated for in the ERFO correction.

The method developed in this paper has been shown to be extremely reproducible. The three test deliveries of each of the six VMAT plans were acquired at monthly intervals, and the results given in Table 1 and 6, 7 indicate that there was no change in MLC performance over a three‐month time period. The consistency of each individual leaf performance is also demonstrated in Fig. 7, which shows the RMS error of each leaf of each bank for a prostate and head and neck plan. The greatest difference in leaf RMS error over this period was less than 0.1 mm.

It can be observed in Table 1 and Fig. 7 that the deviation between the planned and measured MLC positions was higher for the head and neck test plans than for the prostate test plans. This is expected due to the higher level of modulation of head and neck plans which results in a higher average leaf acceleration and leaf speed. Figure 7 also indicates that some leaves perform better than others during the delivery. This is a result of varying levels of complexity in each individual leaf trajectory plan. For this reason, site‐specific tolerances have been established using statistical process control; however, the details of this are not specified in this publication.

Table 2 gives a comparison between the planned dose and the dose reconstructed using EPID‐measured MLC positions. This comparison was performed using a DVH analysis of the PTV and OARs. Only small differences can be seen in the DVH between the planned and reconstructed dose indicating that for these plans the MLC positions are accurate enough to deliver the correct dose distribution to the patient. This demonstrates that, by using this QA tool, decisions regarding MLC positioning accuracy can be made using clinically meaningful volumetric parameters such as DVH statistics.

MLC error detection was demonstrated by introducing different types of errors into VMAT deliveries. The leaf errors introduced included a single leaf calibration errors, leaf pair malfunction error, random leaf calibration errors, and systematic leaf gap errors. As well as geometrically detecting each error, the 3D patient dose was also reconstructed based on the measured MLC positions and compared to the planned dose distribution.

Single‐leaf calibration errors were detected as low as 0.5 mm, as shown in Fig. 8(a). The reconstructed dose for this type of error resulted on only small dose errors in only a small part of the PTV, as shown in Fig. 8(b). This type of error would occur if a single leaf requires recalibration and, even for these small magnitudes, could become significant for small field stereotactic treatments. Single‐leaf miscalibration errors on a small scale may not be detected by other QA methods, such as those which rely on DynaLog files^(14)^ or low‐resolution devices.^25^, ^26^

Random leaf calibration errors may occur when a MLC has not been initialized frequently enough. These types of errors can easily be visualized in Figs. 9(a) and (b) which shows an error histogram for delivery without and with errors respectively. The five peaks in (b) correspond to the magnitude of the discrete random calibration errors that were introduced (i.e., at 0, ±1, and ±2mm). This type of error does not result in any significant dose errors in the patient as seen in Fig. 9(c) and this agrees with previous results regarding random MLC errors in VMAT.^(10)^

Errors in the gap between opposing MLC leaves can result in systematic dose errors in the patient, as demonstrated by Oliver et al.^(10)^ Here the detection of these types of error has been demonstrated for ±1mm and ±2mm gap errors by computing the mean gap error over the entire delivery. Figure 10(a) shows a plot of the measured mean gap error as a function of the actual introduced gap error. The measured MLC positions for the ±2mm cases result in large errors in the PTV and OAR doses as seen in Fig. 10(b).

These results indicate that different types of errors, even with the same magnitude, can have very different levels of significance in terms of the dose errors in the patient. For this reason, rather than analyzing MLC errors simply in terms of positional accuracy, the type of error should also be considered. Furthermore, a particular error will impact each patient differently, depending on which MLCs are involved and the location of the PTV and OARs relative to the time, location, and type of error. This should also be considered when assessing the clinical significance of MLC performance for VMAT deliveries.

Machine log files (DynaLog files) have been used to assess MLC performance during clinical VMAT deliveries. A number of publications have shown that log files can be extremely useful for MLC QA and also for patient‐specific 3D dose reconstruction. However, recent studies have voiced concern for using log files as a primary method for MLC QA. Agnew et al.^(14)^ showed that machine log files were unable to detect systematic errors caused by recalibration requirements, wear and tear of the t‐nut or suboptimal leaf motor performance. The reason for this is that MLC positions from MLC log files are not a measurement of the actual leaf position but are, instead, sourced from the positional encoder of each leaf motor. This means that any type of mismatch between the encoder recorded position and the actual MLC position will not be detected using machine log files. EPID imaging provides a direct measurement of the radiation field edge. This means EPID images can be used as a ground truth for the actual MLC position and are capable of detecting all types of MLC positional errors. The EPID‐based method presented here also has the ability to detect changes in mechanical sag and other gravity‐related effects which would not be reflected in the machine log files but should still be monitored.^27^, ^42^ Future work will be to perform a long‐term comparison between DynaLog files and EPID‐based MLC positions for VMAT deliveries, in order to investigate the advantages and/or disadvantages of using machine log files for routine QA.

While this method is suitable for arc‐resolved MLC QA, collimator angle QA, and gantry motion QA, it does not measure or check that the dose rate throughout the delivery is correct. The 3D dose reconstruction is based on the measured MLC positions but simply assumes that dose fraction as a function of gantry angle is delivered per the delivery plan. This has the advantage of isolating the effect of the MLC performance on the delivered dose but, as a result, it is not an end‐to‐end test of the VMAT delivery and, hence, should not be used as a stand‐alone patient‐specific QA tool. Future work will be to combine this method with a technique for extracting the dose rate from each EPID image frame to independently measure and verify all components of the VMAT delivery from a single measurement.

This system may also be applied in real‐time for *in vivo* MLC trajectory verification. This has been tested using an anthropomorphic pelvic phantom to simulate the patient scatter component of the EPID images. Although this has not yet been demonstrated clinically, processing time for each image frame can currently be achieved within one frame time period (≤120ms) which lends the possibility of real‐time delivery verification using this technique.

## V. CONCLUSIONS

An EPID‐based method was developed and tested to quality assure the positional accuracy of the MLC during VMAT deliveries. MLC trajectories were measured as a function of gantry angle using EPID image frames and were then compared to the planned trajectory of the delivery plan. Using a set of six VMAT plans and three test deliveries of each plan, the MLC extraction method was shown to be reproducible and accurate, yielding a maximum mean error of 0.07 mm and a maximum RMS error of 0.8 mm for any MLC of any delivery. The method was also extended to include a 3D dose reconstruction in the patient using the measured MLC trajectories in order to assess the clinical significance of MLC positional errors. The sensitivity of the system was demonstrated for various types of MLC errors. The software was used to detect each type of error and perform a subsequent dose reconstruction in the TPS to determine the impact of the error on the dose delivery. The method was shown to successfully detect each type of error, including single leaf calibration errors as small as 0.5 mm. This system has the potential to be used for both a routine linac QA and VMAT patient‐specific plan QA for the MLC, and has the ability to relate measured MLC positional errors to 3D dosimetric errors in the patient.

## ACKNOWLEDGMENTS

The authors wish to acknowledge Shashank Bhatia for development of the frame grabber acquisition software used to read and record image frame data from the electronic portal imaging device.

## COPYRIGHT

This work is licensed under a Creative Commons Attribution 3.0 Unported License.
